# B-type natriuretic peptide for the prediction of left ventricular remodelling

**Published:** 2014-02

**Authors:** Christophe Bauters, Marie Fertin, Florence Pinet

**Affiliations:** Centre Hospitalier Régional et Universitaire de Lille, Lille; Inserm U744, Institut Pasteur de Lille, Université de Lille 2, Lille; Faculté de Médecine de Lille, Lille, France; Centre Hospitalier Régional et Universitaire de Lille, Lille; Inserm U744, Institut Pasteur de Lille, Université de Lille 2, Lille; Centre Hospitalier Régional et Universitaire de Lille, Lille; Inserm U744, Institut Pasteur de Lille, Université de Lille 2, Lille

## Dear Sir

We read with great interest the recent article by Choi *et al.*^1^ on the optimal time of B-type natriuretic peptide (BNP) sampling for the prediction of left ventricular (LV) remodelling after myocardial infarction (MI). Indeed, as underscored by Choi *et al.*, LV remodelling remains a significant clinical problem in the modern era of MI management.^2^ In addition, BNP is currently the sole biomarker that has been convincingly associated with LV remodelling in multiple studies (reviewed in Fertin *et al.*^3^). It is therefore important to determine the best window of time for its determination in clinical practice.

Using multivariate analysis in a cohort of 131 patients, the authors found that early levels (two to five days) of BNP were associated with LV remodelling in fully adjusted models, whereas late (three to four weeks) and long-term (six months) levels were not. We previously reported on the usefulness of serial (three to seven days, one, three and 12 months) assessment of BNP to predict LV remodelling after MI in a prospective study of 246 patients with a first anterior Q-wave MI.^4^ Our results, which were at variance from those of the study by Choi *et al.*, demonstrated that BNP levels at any time point were associated with LV remodelling; the association was mild at baseline and stronger during follow up, particularly after three months.

With multivariate analysis, BNP retained its predictive value at one, three and 12 months, but no longer at baseline. These discrepancies between studies may be related to differences in study populations and/or therapeutic management. It is also important to know how missing values were handled when comparing models at different time points. From the data presented by Choi *et al.*, it appears that BNP measurements at six months were lacking in more than 20% of the cases. This could theoretically have ‘disadvantaged’ BNP in late versus early models.

At present, we believe that the optimal timing after MI for BNP determination in clinical practice remains an unsettled question.

## References

[1] Choi H, Yoo BS, Doh JH, Yoon HJ, Ahn MS, Kim JY (2013). The optimal time of B-type natriuretic peptide sampling associated with postmyocardial infarction remodelling after primary percutaneous coronary intervention.. Cardiovasc J Afr.

[2] Savoye C, Equine O, Tricot O, Nugue O, Segrestin B, Sautiere K (2006). Left ventricular remodeling after anterior wall acute myocardial infarction in modern clinical practice (from the REmodelage VEntriculaire [REVE] study group).. Am J Cardiol.

[3] Fertin M, Dubois E, Belliard A, Amouyel P, Pinet F, Bauters C (2012). Usefulness of circulating biomarkers for the prediction of left ventricular remodeling after myocardial infarction.. Am J Cardiol.

[4] Fertin M, Hennache B, Hamon M, Ennezat PV, Biausque F, Elkohen M (2010). Usefulness of serial assessment of B-type natriuretic peptide, troponin I, and C-reactive protein to predict left ventricular remodeling after acute myocardial infarction (from the REVE-2 study).. Am J Cardiol.

